# Reference values of hematological and biochemical parameters in young-adult cynomolgus monkey (*Macaca fascicularis*) and rhesus monkey (*Macaca mulatta*) anesthetized with ketamine hydrochloride

**DOI:** 10.1186/s42826-019-0006-0

**Published:** 2019-07-24

**Authors:** Bon-Sang Koo, Dong-Ho Lee, Philyong Kang, Kang-Jin Jeong, Sangil Lee, Kijin Kim, Youngjeon Lee, Jae-Won Huh, Young-Hyun Kim, Sang-Je Park, Yeung Bae Jin, Sun-Uk Kim, Ji-Su Kim, Yeonghoon Son, Sang-Rae Lee

**Affiliations:** 1 Primate Resources Center, Korea Research Institute of Bioscience and Biotechnology, 351-33, Neongme-gil, Ibam-myeon, Jeongup-si, Jeonbuk 56216 Republic of Korea; 2 National Primate Research Center, Korea Research Institute of Bioscience and Biotechnology, 30 Yeongudanji-ro, Ochang-eup, Chungwon-gu, Cheongju-si, Chungbuk 28116 Republic of Korea; 3 Futuristic Animal Resource & Research Center, Korea Research Institute of Bioscience and Biotechnology, Chungbuk, 28116 Republic of Korea; 4 Department of Functional Genomics, University of Science and Technology, Daejeon, 34113 Republic of Korea

**Keywords:** Non-human primate, Hematology, Biochemistry, Cynomolgus monkey, Rhesus monkey

## Abstract

Nonhuman primate models are valuable in biomedical research. However, reference data for clinical pathology parameters in cynomolgus and rhesus monkeys are limited. In the present study, we established hematologic and biochemical reference intervals for healthy cynomolgus and rhesus monkeys anesthetized with ketamine hydrochloride. A total of 142 cynomolgus monkeys (28 males and 114 females) and 42 rhesus monkeys (22 males and 20 females) were selected and analyzed in order to examine reference intervals of 20 hematological and 16 biochemical parameters. The effects of sex were also investigated. Reference intervals for hematological and biochemical parameters were separately established by species (cynomolgus and rhesus) and sex (male and female). No sex-related differences were determined in erythrocyte-related parameters for cynomolgus and rhesus monkey housed in indoor laboratory conditions. Alkaline phosphatase and gamma glutamyltransferase were significantly lower in females than males in both cynomolgus and rhesus monkeys aged 48–96 months. The reference values for hematological and biochemical parameters established herein might provide valuable information for researchers using cynomolgus and rhesus monkeys in experimental conditions for biomedical studies.

## Introduction

Non-human primates (NHPs) are valid and indispensable animal models for biomedical research because humans and NHPs are similar in behavior, physiology, and organ function. Additionally, the immune systems of NHPs are more similar genetically to humans than other animal models such as rodents, rabbits, and dogs. For example, rodents, who are evolutionary and genetically distant from humans, share only 64% genome identity with humans [1], while macaques and humans are 92% genetically similar [2]. The cynomolgus (*Macaca fascicularis*) and the rhesus macaque (*Macaca mulatta*) are the most widely used NHP models in various biomedical research fields, including genomic analysis [3] and neurodegenerative disease [4, 5], as well as reproductive biomedical research [6–8].

As a result of the increased use of cynomolgus and rhesus monkeys in biomedical research, it is necessary to establish the hematological and biochemical parameters of these monkey species. Reference values are imperative to support the selection of healthy animals and to interpret laboratory data in NHP models. Studies have shown differences in blood hematology and biochemistry for laboratory cynomolgus and rhesus macaques according to origin, age, sex, and species [9–11]. Comparative blood values were reported between cynomolgus and rhesus monkeys [9]. Other report have demonstrated that some biochemical and hematological analytes were significantly influenced by sex in cynomolgus from Mauritius [10]. In addition, previous studies have reported that hematological and biochemical parameters can vary due to pre-analytical and environmental conditions [10, 12]. Previous report also investigated that the alterations of hematological and serum biochemical values can be found in animals with ketamine anesthesia [12]. However, despite the importance of non-human primates in biomedical research, only a few studies have examined the reference intervals of hematology and biochemistry for cynomolgus and rhesus macaques kept under laboratory conditions. Therefore, the aim of this study was to provide comprehensive and accurate reference intervals of hematological and biochemical values for evaluating individual health condition indices of individually housed cynomolgus and rhesus macaques prior to their use in biomedical studies.

## Materials and methods

### Animals

The study population was composed of 142 cynomolgus monkeys (28 males and 114 females; age: 48–96 months) and 42 rhesus monkeys (22 males and 20 females; age: 48–96 months) obtained from China using a Convention on International Trade in Endangered Species of Wild Fauna and Flora permit. All experimental animals were considered “young-adult,” according to the age classification standards for humans to macaques [13–15]. The monkeys in this study were housed in individual cages with sliding horizontal divider in the National Primate Research Center (NPRC) at the Korea Research Institute of Bioscience and Biotechnology (KRIBB). The monkeys were housed in stainless-steel cages (600 W × 800 L × 800H mm) and the environment was controlled to provide a temperature of 23 ± 1 °C, a relative humidity of 50 ± 5%, and a 12:12 h light-dark cycle. Animals were fed twice daily with fresh fruits and commercial food intended for laboratory primates (Harlan primate diet, USA), and water was available ad libitum. All monkeys were determined to be healthy by history and veterinary examination and all procedures were approved by the animal care and use committee of KRIBB (Approval No. KRIBB-AEC-11010, 15031, 16067). In addition, all experiments were conducted in compliance with national guidelines and complying with the Guide for the Care and Use of Laboratory Animals [16]. Hematology and biochemistry data reported in the present study were derived during NPRC health monitoring at KRIBB between 2014 and 2016. No animals were sacrificed in this study.

### Blood sampling and preparation

After overnight fasting (14–16 h), animals were deeply anesthetized with ketamine (5 mg/kg) by intramuscular injection, and 4-ml blood samples were drawn from femoral veins using 23-gauge needles. Then, 1-ml aliquots from all blood samples were individually transferred into dipotassium ethylenediaminetetraacetic acid (EDTA-K2) tubes for hematological analysis, and the remaining 3-ml aliquots were transferred into heparin tubes, where plasma was separated by centrifugation at 1600 g for 15 min for biochemical analysis. All samples were processed within 2 h of blood draw.

### Hematological and biochemical analysis

Prior to hematological testing, each sample was gently homogenized on a blood tube roller mixer for 15 min. Hematology measurements were performed on whole blood using a HEMAVET 950FS hematology analyzer (Drew Scientific, Cumbria, UK). Biochemical analysis was carried out using a Dri-Chem 7000i biochemistry analyzer (Fujifilm, Tokyo, Japan). Parameters, abbreviations, and units of hematological and biochemical values are listed in Table 1.

**Table 1:** Abbreviations, units of measure, methods for hematological and biochemical parameters

Parameter	Abbreviation	Unit
Hematology		
Red blood cell	RBC	10^6^/μL
Hemoglobin	Hb	g/dL
Hematocrit	HCT	%
Mean corpuscular volume	MCV	fL
Mean corpuscular hemoglobin	MCH	pg
Mean corpuscular hemoglobin concentration	MCHC	g/dL
Red blood cell volume distribution width	RDW	%
White blood cell	WBC	10^3^/μl
Neutrophil	Neut	10^3^/μl
Neutrophil percentage	Neut	%
Lymphocyte	Lymph	10^3^/μl
Lymphocyte percentage	Lymph	%
Monocyte	Mono	10^3^/μl
Monocyte percentage	Mono	%
Eosinophil	Eosin	10^3^/μl
Eosinophil percentage	Eosin	%
Basophil	Baso	10^3^/μl
Basophil percentage	Baso	%
Platelet	PLT	10^3^/μl
Mean platelet volume	MPV	fL
Biochemistry		
Aspartate aminotransferase	AST	U/l
Alanine aminotransferase	ALT	U/l
Alkaline phosphatase	ALP	U/l
Blood urea nitrogen	BUN	mg/dl
Creatinine	CRE	mg/dl
Glucose	GLU	mg/dl
Total cholesterol	TCHO	mg/dl
Albumin	ALB	g/dl
Total protein	TP	g/dl
Triglyceride	TG	mg/dl
Total bilirubin	TBIL	mg/dl
Inorganic phosphorus	IP	mg/dl
Calcium	Ca	mg/dl
Gamma glutamyltransferase	GGT	U/l
Lactate dehydrogenase	LDH	U/l
Direct bilirubin	DBIL	mg/dl

### Statistical analysis

Data are reported as mean ± SD. The results of hematological and biochemical parameters were analyzed using two-tailed Student’s t-tests (Graphpad Prism 6) in order to detect significant differences between the male and female members of each species. In all analyses, a *p-*value less than 0.05 was deemed to indicate statistical significance.

## Results

### Reference ranges of hematological and biochemical parameters in cynomolgus and rhesus monkeys

Reference ranges of hematological and biochemical parameters are reported in Tables 2, 3, 4 and 5. Tables 2 and 3 list the reference values in cynomolgus and rhesus monkeys, respectively, for red blood cell (RBC) count, hemoglobin (Hb), hematocrit (HCT), mean corpuscular volume (MCV), mean corpuscular hemoglobin (MCH), mean corpuscular hemoglobin concentration (MCHC), red cell distribution width (RDW), white blood cell (WBC) count, neutrophil (Neut) count, lymphocyte (Lymph) count, monocyte (Mono) count, eosinophil (Eosin) count, basophil (Baso) count, percent WBC for differential counts, platelet (PLT) count, and mean platelet volume (MPV). Tables 4 and 5 include the results for levels of aspartate aminotransferase (AST), alanine aminotransferase (ALT), alkaline phosphatase (ALP), blood urea nitrogen (BUN), creatinine (CRE), glucose (GLU), total cholesterol (TCHO), albumin (ALB), total protein (TP), triglyceride (TG), total bilirubin (TBIL), inorganic phosphate (IP), calcium (Ca), gamma glutamyl transferase (GGT), lactate dehydrogenase (LDH), and direct bilirubin (DBIL) in cynomolgus and rhesus macaques, respectively

**Table 2:** Hematological parameter values of cynomolgus monkeys aged 48–96 months

Parameter (unit)	Males (*n* = 28)	Females (*n* = 114)	Male range (*n* = 28)	Female range (*n* = 114)
RBC (10^6^/μl)	5.40 ± 0.36	5.30 ± 0.51	4.73–6.46	4.16–7.32
Hb (g/dl)	11.6 ± 1.05	11.1 ± 1.16	10.1–14.8	9.10–17.00
HCT (%)	40.4 ± 3.17	38.4 ± 3.87*	35.6–49.2	31.6–57.2
MCV (fl)	74.8 ± 3.91	72.6 ± 3.94*	68.0–83.5	63.8–82.9
MCH (pg)	21.5 ± 1.45	21.1 ± 1.18	18.4–23.9	17.1–24.2
MCHC (g/dl)	28.8 ± 1.15	29.1 ± 1.16	24.4–30.1	23.6–33.9
RDW (%)	13.9 ± 0.73	14.2 ± 0.68	12.5–15.6	12.9–16.4
WBC (10^3^/μl)	7.46 ± 2.12	7.86 ± 2.74	3.88–11.3	3.04–18.4
Neut (10^3^/μl)	4.30 ± 1.88	4.33 ± 2.22	1.49–8.6	1.07–14.9
Neut (%)	57.2 ± 18.5	54.3 ± 13.8	30.52–88.24	18.6–82.0
Lymph (10^3^/μl)	2.71 ± 1.45	3.05 ± 1.40	0.59–5.33	0.85–7.87
Lymph (%)	36.7 ± 17.5	39.6 ± 13.1	8.64–61.13	12.3–70.4
Mono (10^3^/μl)	0.19 ± 0.11	0.19 ± 0.10	0.03–0.50	0.03–0.66
Mono (%)	2.50 ± 1.16	2.49 ± 1.02	0.56–6.06	0.65–5.87
Eosin (10^3^/μl)	0.25 ± 0.10	0.27 ± 0.11	0.08–0.47	0.09–0.65
Eosin (%)	3.44 ± 1.47	3.55 ± 1.13	1.04–9.58	1.55–6.91
Baso (10^3^/μl)	0.01 ± 0.01	0.01 ± 0.01	0.00–0.04	0.00–0.06
Baso (%)	0.14 ± 0.14	0.10 ± 0.10	0.00–0.52	0.00–0.64
PLT (10^3^/μl)	410 ± 104	437 ± 78.4	233–712	281–672
MPV (fl)	10.8 ± 1.42	11.5 ± 1.64	8.90–14.8	7.60–16.3

**Table 3:** Hematological parameter values of rhesus monkeys aged 48–96 months

Parameter (unit)	Males (*n* = 22)	Females (*n* = 20)	Male range (*n* = 22)	Female range (*n* = 20)
RBC (10^6^/μl)	5.39 ± 0.66	5.27 ± 0.67	4.39–7.02	3.85–6.63
Hb (g/dl)	11.4 ± 1.35	11.6 ± 1.37	9.60–14.3	9.20–14.3
HCT (%)	37.8 ± 5.10	39.5 ± 5.07	25.5–46.9	30.1–52.0
MCV (fl)	72.2 ± 2.94	75.0 ± 4.01*	67.6–77.5	68.7–83.5
MCH (pg)	21.2 ± 1.59	22.0 ± 1.37	18.7–26.0	20.0–24.8
MCHC (g/dl)	29.5 ± 2.33	29.4 ± 1.58	25.7–36.9	27.2–33.7
RDW (%)	13.9 ± 0.71	14.0 ± 0.71	12.7–15.2	12.8–15.4
WBC (10^3^/μl)	6.31 ± 2.62	7.36 ± 1.79	3.10–12.1	4.60–11.1
Neut (10^3^/μl)	3.37 ± 1.95	3.75 ± 1.04	1.29–8.90	2.19–5.43
Neut (%)	52.3 ± 15.2	51.6 ± 11.4	34.2–87.9	33.1–72.9
Lymph (10^3^/μl)	2.43 ± 1.29	3.02 ± 1.29	0.44–5.45	1.43–6.51
Lymph (%)	39.8 ± 14.5	40.4 ± 11.2	5.49–59.4	20.6–61.4
Mono (10^3^/μl)	0.17 ± 0.13	0.22 ± 0.12	0.06–0.52	0.07–0.58
Mono (%)	2.75 ± 1.40	2.94 ± 1.17	0.75–6.15	0.95–5.39
Eosin (10^3^/μl)	0.33 ± 0.18	0.36 ± 0.17	0.09–0.67	0.13–0.72
Eosin (%)	5.13 ± 1.89	4.94 ± 2.07	1.48–8.60	2.03–9.49
Baso (10^3^/μl)	0.00 ± 0.01	0.01 ± 0.01	0.00–0.02	0.00–0.02
Baso (%)	0.08 ± 0.07	0.10 ± 0.07	0.00–0.27	0.00–0.27
PLT (10^3^/μl)	321 ± 100	346 ± 57.9	155–619	260–461
MPV (fl)	10.9 ± 1.67	11.5 ± 2.40	8.00–14.8	9.20–18.1

**Table 4:** Biochemical parameter values of cynomolgus monkeys aged 48–96 months

Parameter (unit)	Males (*n* = 28)	Females (*n* = 114)	Male range (*n* = 28)	Female range (*n* = 114)
AST (U/l)	33.6 ± 24.5	31.9 ± 15.3	15.0–114	13.0–106
ALT (U/l)	8.70 ± 6.92	11.8 ± 10.9	1.00–29.0	1.00–56.0
ALP (U/l)	667 ± 337	376 ± 133***	188–1670	158–741
BUN (mg/dl)	18.6 ± 5.37	16.1 ± 4.23*	7.20–30.7	7.50–28.0
CRE (mg/dl)	0.94 ± 0.27	0.71 ± 0.20***	0.60–1.60	0.10–1.10
GLU (mg/dl)	67.5 ± 18.5	67.4 ± 16.4	33.0–120	26.0–130
TCHO (mg/dl)	105 ± 27.8	123 ± 31.4*	52.0–164	37.0–203
ALB (g/dl)	3.87 ± 0.49	3.85 ± 0.38	2.90–4.90	2.20–4.80
TP (g/dl)	6.68 ± 0.66	6.72 ± 0.60	5.40–7.60	4.60–8.10
TG (mg/dl)	42.4 ± 37.6	46.4 ± 28.0	8.00–215	9.00–213
TBIL (mg/dl)	0.27 ± 0.18	0.34 ± 0.26	0.10–0.08	0.10–2.30
IP (mg/dl)	4.35 ± 1.14	3.74 ± 1.10*	1.20–6.90	1.10–6.60
Ca (mg/dl)	8.30 ± 0.82	8.44 ± 0.67	6.80–9.90	6.20–9.90
GGT (U/l)	83.4 ± 33.1	63.7 ± 26.2**	35.0–181	1.00–199
LDH (U/l)	268 ± 198.8	194 ± 100.7**	53.0–900	31.0–467
DBIL (mg/dl)	0.13 ± 0.06	0.19 ± 0.12*	0.10–0.30	0.10–0.80

**Table 5:** Biochemical parameter values of rhesus monkeys aged 48–96 months

Parameter (unit)	Males (*n* = 22)	Females (*n* = 20)	Male range (*n* = 22)	Female range (*n* = 20)
AST (U/l)	25.7 ± 10.3	20.0 ± 8.55	12.0–55.0	7.00–42.0
ALT (U/l)	9.80 ± 5.87	8.20 ± 6.12	1.00–29.0	1.00–22.0
ALP (U/l)	629 ± 320	386 ± 192***	42.1–1635	146–791
BUN (mg/dl)	15.3 ± 3.80	15.0 ± 3.62	10.2–22.6	9.30–23.7
CRE (mg/dl)	0.89 ± 0.18	0.82 ± 0.16	0.60–1.20	0.50–1.10
GLU (mg/dl)	74.0 ± 20.7	81.1 ± 18.1	33.0–120	55.0–126
TCHO (mg/dl)	112 ± 32.5	129 ± 26.5	17.0–170	74.0–182
ALB (g/dl)	4.50 ± 0.33	4.30 ± 0.58	3.90–5.10	3.10–5.20
TP (g/dl)	6.70 ± 0.81	7.32 ± 0.84**	3.90–7.80	5.20–8.70
TG (mg/dl)	41.9 ± 26.2	44.3 ± 16.6	7.40–134	19.0–78.0
TBIL (mg/dl)	0.27 ± 0.12	0.20 ± 0.10*	0.10–0.70	0.10–0.40
IP (mg/dl)	4.55 ± 0.79	4.37 ± 0.80	3.30–6.60	3.10–6.00
Ca (mg/dl)	8.35 ± 0.77	8.36 ± 0.92	7.00–9.80	6.40–9.50
GGT (U/l)	84.8 ± 27.7	66.8 ± 24.8*	2.00–128	24.0–130
LDH (U/l)	136 ± 58.9	130 ± 64.4	59.0–287	33.0–224
DBIL (mg/dl)	0.19 ± 0.14	0.20 ± 0.10	0.10–0.50	0.10–0.40

### Effect of sex on hematological values in cynomolgus and rhesus monkeys

Hematological analysis results are presented in Tables 2 and 3. No significant changes was found on RBC count both in cynomolgus (Table 2) and rhesus monkeys (Table 3). In terms of the effects of sex, HCT and MCV values were significantly higher in the male cynomolgus monkeys than in the female monkeys. In the rhesus monkeys, MCV showed significantly lower values in males than in females. WBC counts tended to increase in female compared to male macaques, although there were no significant differences. In addition, no significant changes were observed in PLT parameters in both cynomolgus and rhesus monkeys.

### Effect of sex on biochemical values in cynomolgus and rhesus monkeys

The results obtained from analyzing biochemical parameters are listed in Tables 4 and 5. ALP and GGT values were significantly higher in males than in females in both cynomolgus and rhesus macaques (Tables 4 and 5). Significant sex-related differences were noted for BUN and CRE in cynomolgus monkeys. In addition, IP and LDH concentrations were found to be significantly higher levels in male than female cynomolgus monkeys. Moreover, significant effects by sex were also shown for TCHO, and DBIL in cynomolgus monkeys aged between 48 and 96 months. In rhesus monkeys, sex had a statistically significant effect on TP and TBIL.

## Discussion

In the present study, the biochemistry and hematology of young-adult NHPs (cynomolgus and rhesus macaques) housed in indoor individual cages were analyzed to establish reference indices for several parameters. In addition, we pointed to fasting, sedation of the monkeys, which might be one of the important factor of variation.

Cynomolgus and rhesus monkeys both belong to the macaque family and have been increasingly used in various biomedical research fields. Due to the close relationship between humans and macaques, the cynomolgus and rhesus monkey, the most commonly used non-human primates in medical research, serves as a valuable animal model in neuroscience, aging disease, reproductive physiological, and behavioral research [17]. Previous studies have reported reference values for clinical chemistry and hematology parameters in cynomolgus monkeys [11, 18] as well as in rhesus macaques [19, 20]. However, large discrepancies exist in the reported results, which might be influenced by age [11], sex [10], species [15], sedation, or fasting before blood sampling [12]. Therefore, it is essential to establish proper reference values to assess health condition, biological variation, or the effects of drugs or endocrine-disrupting chemicals on the basis of each environmental system.

One of the potential sources of variation in this study were the fasting prior to blood sampling. Due to the housing conditions, we were able to assure that all monkeys were strictly fasted overnight, because food remains were retrieved after the last feeding schedule. Although it has been previously reported that fasting is not an absolute prerequisite in hematology [21], it could be clinically relevant in monkeys if the fasting duration exceeds 16 h [22]. Therefore, about 16-h fasting duration used in this study might be clinically relevant reference values for NHPs.

Another pre-analytical variable in this study was the sedation of animals with ketamine prior to blood sampling. A previous study demonstrated that ketamine anesthesia reduces leukocyte count in cynomolgus monkeys [23]. Further, reduced leukocyte counts have been observed in rhesus monkeys with ketamine sedation [24]. It has been reported that ketamine anesthesia significantly reduces WBC counts and lymphocyte percentages in cynomolgus monkeys compared to non-anesthetized animals [12]. In the present study, WBC counts were relatively low compared to previous studies [12, 19], and there was a slight elevation in the subpopulation of neutrophils, which might be caused by the potentially stressful environmental conditions (e.g. individual cages).

Sex-related differences were expected in hematology and biochemistry analytes. It has been previously reported that males show significantly higher values for RBC count, Hb, and HCT in cynomolgus monkey [11, 25] as well as human [26], which might be related to menstruation-related blood loss. However, other studies have reported no effect by sex on any red blood cell analytes [18, 21]. In the present study, there were no marked differences in erythrocyte levels between females and males in either cynomolgus or rhesus macaques. These discrepancies might be due to the differences in the reference sample group (24–36 month-olds vs 48–96 month-olds) or environmental conditions (indoor vs outdoor). Sex-associated differences have also been reported in various biochemical parameters. One previous study reported that ALP concentrations were lower in females than males in cynomolgus monkeys who were over 37 months in age [11]. Previous study also reported that the mean levels of ALP concentrations were significantly higher in boys than girls, indicating that similar changes in ALP was also reported in humans [27]. In addition, sex-related difference in GGT levels have been demonstrated in cynomolgus monkeys [28]. Likewise, we observed that the concentrations of ALP and GGT were significantly higher in males than in females in both cynomolgus and rhesus monkeys aged between 48 and 96 months.

In conclusion, we focused our analysis in order to establish baseline values of hematological and biological parameters in two species of NHPs (cynomolgus and rhesus monkeys) with experimental conditions. Because the pre-analytical and analytical conditions were notably different, it is difficult to compare the observed values in the present study with those in the literature. However, the reference ranges of hematological and biological parameters established in the present study might be representative of a reference population of young-adult cynomolgus and rhesus macaques housed in stressing conditions and may therefore serve as a basis for selecting healthy animals and evaluating preclinical studies. Further comprehensive studies are required to explore the effect of age and sex on physiological parameters in animals with other ages, including infant to juvenile (0–5 years), middle-aged adults (10–20 years), and old adults (20+ years).

## Data Availability

The datasets generated or analysed during this study are included in this published article.
